# Big data in contemporary electron microscopy: challenges and opportunities in data transfer, compute and management

**DOI:** 10.1007/s00418-023-02191-8

**Published:** 2023-04-13

**Authors:** David Poger, Lisa Yen, Filip Braet

**Affiliations:** 1 Microscopy Australia, The University of Sydney, Sydney, NSW 2006 Australia; 2 Australian Centre for Microscopy and Microanalysis, The University of Sydney, Sydney, NSW 2006 Australia; 3 School of Medical Sciences (Molecular and Cellular Biomedicine), The University of Sydney, Sydney, NSW 2006 Australia

**Keywords:** Best practices, FAIR and CARE principles, Metadata standards, Microscopy facility, Virtual research environment, Workflow optimisation

## Abstract

The second decade of the twenty-first century witnessed a new challenge in the handling of microscopy data. Big data, data deluge, large data, data compliance, data analytics, data integrity, data interoperability, data retention and data lifecycle are terms that have introduced themselves to the electron microscopy sciences. This is largely attributed to the booming development of new microscopy hardware tools. As a result, large digital image files with an average size of one terabyte within one single acquisition session is not uncommon nowadays, especially in the field of cryogenic electron microscopy. This brings along numerous challenges in data transfer, compute and management. In this review, we will discuss in detail the current state of international knowledge on big data in contemporary electron microscopy and how big data can be transferred, computed and managed efficiently and sustainably. Workflows, solutions, approaches and suggestions will be provided, with the example of the latest experiences in Australia. Finally, important principles such as data integrity, data lifetime and the FAIR and CARE principles will be considered.

## Introduction

Electron microscopy (EM) of biological samples has changed dramatically since the development of the first electron microscopes in the 1930s. Over the past 10 years, the rapid advances in EM, especially in cryogenic electron microscopy (cryo-EM), cryogenic electron tomography (cryo-ET), volume electron microscopy (VEM) and correlative light and electron microscopy (CLEM), have enabled the collection of high-volume, high-complexity and high-resolution data at an ever-increasing speed (Hauser et al. 2017; Ando et al. 2018; Danev et al. 2019; Eisenstein 2023). Advances in cryo-EM were indeed recognised in 2017 with the Nobel Prize in Chemistry. Progress in sample preparation, instrumentation and algorithms for image processing has continually pushed the boundaries of possibilities (Kühlbrandt 2014; Bai et al. 2015; Schur 2019; Chua et al. 2022). Cryo-EM, cryo-ET, VEM and CLEM are now capable of resolving unprecedented, near-atomic-resolution structures of single proteins and molecular complexes (Bai et al. 2013; Nakane et al. 2020; Yip et al. 2020; Cao et al. 2021; Lazić et al. 2022) but also aggregates, self-assemblies, whole cells and molecular sociology of cells at near-atomic detail (Beck and Baumeister 2016; Oikonomou and Jensen 2017; Guo et al. 2018; Shahmoradian et al. 2019; Bäuerlein and Baumeister 2021).

As EM embraces its “resolution revolution” (Kühlbrandt 2014), new challenges have emerged. Nowadays, EM experiments and subsequent data processing and analysis yield ever more data that require dedicated techniques, technologies and infrastructure suitable for data-intensive science. For example, specialised hardware for compute and optimised software for data transfer have become critical to manage this “data deluge” (Bell et al. 2009). However, the nature of this explosion of data cannot be reduced to large amounts of data. EM has shifted towards big data. The concept of “big data” has been given many definitions, but, in general, big data are associated with at least three attributes: high volume, high velocity and high variety (Assunção et al. 2015; Hilbert 2016). Alongside the resolution revolution, the big-data revolution necessitates new paradigms to process and manage data in order to optimise tasks and enable discovery. The ability to extract value from big data is key and depends on data analytics that turns data into insights (Jagadish et al. 2014; Assunção et al. 2015). This is essential, as otherwise instead of a big-data revolution rich in new opportunities, it could become a big-data flood that prevents realisation of the maximum value from EM big data. As EM underwent its big-data revolution, it expanded its user base in a very short time from a small number of experts to a bigger and more diverse community of users, making cryo-EM and cryo-ET mainstream methods for structural biology alongside X-ray crystallography and nuclear magnetic resonance spectroscopy.

Finally, as a last challenge, the big-data revolution in EM has unfolded at a time when there has been a strong drive to make scientific data findable, accessible, interoperable and reusable (FAIR) (Wilkinson et al. 2016). In essence, the FAIR principles require that research data and metadata, as well as the workflows, tools and repositories that they are associated with, foster knowledge discovery, experiment reproducibility and research impact by assisting humans and machines in their discovery, access, sharing and integration with other data and applications for processing, analysis and storage. The FAIR principles put specific emphasis on machine actionability, that is, enhancing the ability of computational systems to automatically find, access, interoperate and reuse data with no or minimal human intervention. This is especially relevant in the current explosion of data in science. In addition, and complementary to the FAIR principles, the CARE Principles for Indigenous Data Governance (collective benefit, authority to control, responsibility and ethics) have been proposed to provide guidance to data producers, users, managers and publishers on the inclusion of Indigenous Peoples in data processes that strengthen indigenous control for improved discovery, access, use, reuse and attribution in contemporary data landscapes (Carroll et al. 2020). As the FAIR and CARE principles permeate through all fields of research, addressing the challenges of big data in light of those principles gives the unique opportunity to change current practices and promote best ones amongst researchers and research facilities in the way that they handle EM data from capture through to storage, sharing and disposal.

Many fields of research – including particle physics (Britton and Lloyd 2014; Klimentov et al. 2015), astronomy (Kremer et al. 2017), chemical engineering (Chiang et al. 2017), climatology (Schnase et al. 2017), genomics (Palacio and López 2018) and synchrotron science (Wang et al. 2018) – have experienced their own big-data revolution and faced similar challenges to EM. The sheer volumes and complexity of data generated at the time of capture by electron microscopes and during data processing and analysis have posed new problems to and created opportunities for both researchers and research facilities that operate microscopes. Transferring, storing, sharing, processing and analysing data have become far from trivial in the era of big-data EM, but there are approaches available to harness the power of big data. In this review, we will outline what we understand about big data in the EM sciences, alongside the challenges and opportunities that big data present to both researchers and microscopy research facilities. This thematic paper discusses approaches available for big-data transfer, processing, analysis and management. In particular, advances in software, hardware and workflows – which, together, form the key infrastructure underpinning contemporary research facilities and electron microscopes – are outlined in the context of the expansion of the EM user base and the promotion of the FAIR and CARE principles. We will use our experiences on big data in cryo-EM as an example case. However, those insights are also directly applicable to VEM and CLEM and are approached throughout the paper. Note, a workflow in this review is understood as a series of tasks or steps in a process to accomplish an objective (Ludäscher et al. 2009).

## Big data in electron microscopy

The concept of “big data” originated in the business and information-technology sectors around the 1990s to early 2010s period (Kitchin 2014). It has since gained in popularity across business circles and media, as well as in the scientific community. This has sparked many debates on its precise definition, so much so that it has become a wide-ranging term for which a unified definition across all business sectors and scientific disciplines has been a moving target. There is nevertheless a consensus that big data are characterised by three properties often dubbed “the three V’s”: volume, variety and velocity (Assunção et al. 2015; Hilbert 2016). Volume defines the amounts of data. Variety corresponds to the range of data types (such as structured and unstructured data; data formats; small and large files) and sources. Velocity refers to the speed of growth of data or the speed at which data arrive or are produced at various stages in workflows (upstream at data collection or creation, or downstream during data processing or analysis). Importantly, what data volume, variety and velocity encompass in the scientific community in general, and in microscopy in particular, may differ from traditional views in information technology (IT).

### The properties of big data

While volume is often considered the key and most evident feature of big data, what constitutes large data volumes may vary with institution, discipline, technique, image-acquisition settings or intrinsic ability of infrastructure (hardware, software, workflow) to support an instrument. For example, the scalability (or lack thereof) of existing storage hardware may be an asset (or a hindrance) to deal with increasing data volumes. What separates “big and challenging” from “small and manageable” data may be consequently arbitrary. Big data are thus not defined by specific size or speed metrics but rather by the fact that they cannot be managed by traditional processes and tools due to their size, velocity or variety (Miele and Shockley 2013).

Variety in big data encompasses the diversity in data types (models and formats) and sources. This includes the possible incompatibility between data formats or format versions and the lack of interoperability of data formats and software applications, as well as the ability to maintain and support various versions of software packages or tools associated with the data. The heterogeneity of sources of data may be broadened in science beyond the common definition in IT that limits the variety of sources to elements such as texts, audio, video, web pages, social media and reports (Miele and Shockley 2013). A range of diverse factors contribute to the variety of sources in science, making datasets complex and challenging to combine and manage (Chiang et al. 2017; Richarz 2020). Examples include different techniques (e.g. microscopy, mass spectrometry and omics), inconsistent data models and formats (e.g. lists of scalar values, one- or multi-dimensional arrays), inconsistent conventions used for metadata for data annotation or description, inconsistent modalities to access data, the difficulty to integrate or collate data due to their scattering across geographical locations or storage systems (including instrument or operator log books, laboratory notebooks and electronic laboratory notebooks), and different requirements in data management (for example, regarding data governance).

Finally, there are also several dimensions in data velocity. The reception of incoming data and the creation of outgoing data at any point along a workflow occur at different rates and can be performed following four modes: (1) in batches (data points are grouped together and released for processing at regular time intervals); (2) in near-real time (at small regular time intervals); (3) in real time (continuous input, processing and output of data in a steady flow while retaining the ability to stop or adjust processing to adapt to changes such as incorrect, artefactual or biased input or output data); or (4) in stream (data flow through processing regardless of data quality). Some steps in a workflow may have different velocity requirements: while some software applications may be compatible with more than one of these four modes, other applications may run in one specific mode only. Velocity also poses challenges to transferring big data. For example, transmitting data through a workflow rapidly or data that grow rapidly may require changes in the software or network infrastructure used to transfer big data.

Besides the three V’s (i.e. volume, variety and velocity), a range of other attributes of big data have been discussed in the literature in relation to specific challenges for the management (such as data transfer, storage, sharing and archiving), analysis or visualisation of big data (Miele and Shockley 2013; Assunção et al. 2015; Gandomi and Haider 2015; Khan et al. 2019). Two of those extra properties are often cited as core properties alongside the three V’s: data veracity and data value. First, data veracity relates to the trustworthiness of data and to the degree of uncertainty and inaccuracy associated with data. High data quality underlies reliability of big data (Mehnert et al. 2019). Although there are factors that are unpredictable or difficult to predict that can alter the quality of data and as a result their level of reliability, specific procedures in data cleansing ensure that datasets can be trusted by removing or fixing incorrect, incomplete or corrupted data, as well as duplicated or improperly formatted data (Miele and Shockley 2013). While data cleansing is not specific to big data, traditional methods used so far by researchers may not scale up. Secondly, data value is often considered amongst the most important properties of big data. It corresponds to the usefulness, potential or adequacy of the data to contribute to the research project or the business (Richarz 2020). Value also takes financial considerations, such as the cost to collect, analyse, store and archive data (Khan et al. 2018; Richarz 2020). A range of diverse factors have a direct and great impact on value, including: data governance; best practices, ethical research practices, community standards and conventions such as the FAIR and CARE principles (Wilkinson et al. 2016; Carroll et al. 2020); but also what researchers, research funders and publishers may consider valuable, that is, sound, rigorous, reproducible, publishable or fundable research. In the case of data governance, policies and procedures that determine and maintain the level of availability, accessibility, quality, integrity and security of data from their creation to their disposal or archiving contribute to the valuation of data. Therefore, unlike volume, variety, velocity and veracity, the value attributed to data can change over time and across research organisations and disciplines.

### The ten V’s of big data in electron microscopy

**Fig. 1:**
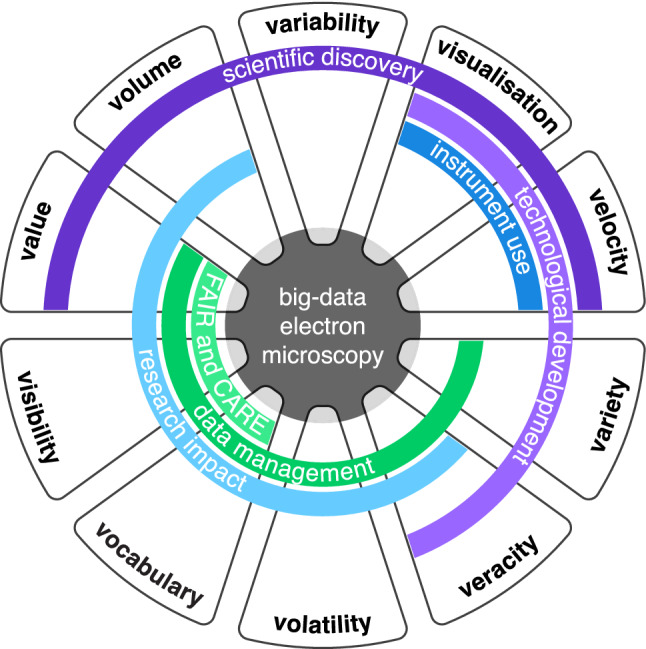
Illustration of the opportunities offered by the ten V’s of big data in electron microscopy

As science embraces big data, a range of disciplines have explored what big data mean to them, in terms of both challenges and opportunities, for example in medicine (Salathé 2016), chemical engineering (Chiang et al. 2017), ecology (Farley et al. 2018) and toxicology (Richarz 2020). The focus of science fields on data volume, variety, velocity and other properties has not been uniform across all fields as the relevance and the pressing nature of the challenges constituted by some of those properties are differently perceived. After reviewing the above literature, including our project findings, Table 1 lists the ten attributes of big data that we consider worth considering in EM because of their importance, namely: volume, variety, velocity, veracity, value, visibility, visualisation, vocabulary, variability and volatility. Of special note, this list is arbitrary and additional properties of big data such as validity (suitability of data to a specific model or application), viscosity (data complexity), verification (data authenticity and desired outcome) and vulnerability (data security) (Khan et al. 2019) also apply to EM but were not included. Importantly, despite the challenges presented by the ten V’s of big data in EM, they also represent multiple opportunities across a range of domains (Fig. 1): scientific discovery (data volume, velocity, value, visualisation and variability), technological development in hardware and software (data variety, velocity, veracity and visualisation), optimised use of instruments (data velocity and visualisation), enhanced research impact (data volume, value, veracity, visibility, vocabulary and volatility), implementation of best practices in data management (data variety, veracity, value, visibility, vocabulary and volatility) and the operationalisation of the FAIR and CARE principles (data value, visibility and vocabulary). None of the challenges and opportunities is indeed new. The big-data era of EM is at the confluence of technological advances in instrumentation, development in hardware, software and algorithm for data processing and analysis, and widespread promotion of best practices in data management across research to put the FAIR and CARE principles in practice. Consequently, workflows, tools and methods that traditionally worked well at smaller scales to manage and analyse EM data (until about 2015) are currently being urgently revisited to address issues related to big data while considering the evolution of the research environment and efficient use of instruments. In this review, a range of approaches to the transfer, processing, analysis and management of EM big data are outlined to demonstrate that suitable approaches are available or being developed to address the challenges posed by the ten V’s of big data in EM and exploit the opportunities created.

**Table 1:** The ten V’s of big data in electron microscopy with their respective challenges versus opportunities

Property (“V”)	Description	Challenge	Opportunity
Volume	Large amount of data created	Storage, transfer, processing and analysis of data; cleansing, annotation, description and curation of data	Increased time or space resolution; large training set for modelling and prediction (e.g. artificial intelligence); greater statistical significance; meta-analysis
Variety	Diverse range of data types (models and formats), sizes and sources	Variations in the efficiency of data transfer; management of many different proprietary and non-proprietary data formats; various data sources	Each data type may include additional information that standardised formats may not contain
Velocity	Rate at which data are created or processed	Transferring and storing data at a high-enough speed compatible with the data creation or processing rate	Near-real-time or real-time data processing, analysis or visualisation to allow fast data quality assessment and optimisation of instrument time
Veracity	Confidence, trust in or reliability of data	Assessment of data quality; uncertainty in data quality, reliability or accuracy; difficulty in annotating or describing data	Robust data cleansing procedures; large volumes of high- or average-quality data can compensate for smaller volumes of low-quality data; description of how data were acquired, processed and analysed
Value	Usefulness, potential or adequacy of data to contribute to the research undertaken	Large data volumes may make extracting relevant, meaningful and useful information more difficult; data value can change	Large data volumes may increase the chance of discovery; FAIR and CARE data have a greater value; standardisation of data formats enhances data value
Visibility	Availability, findability, accessibility and sharing of data under well-defined conditions (FAIR and CARE data), or no limitation (open data)	Sustainability of data repositories	Greater research impact through increased visibility of data in repositories; clarity and transparency around the conditions governing data access and sharing
Visualisation	Graphical representation of data	Traditional visualisation programs may have limitations regarding big data (e.g. memory, response time)	Near-real-time or real-time data visualisation for sample screening, data-quality assessment and optimisation of instrument time
Vocabulary	Schema, data models, ontologies, classifications and metadata that describe and define the structure, syntax, content and provenance of data, and relationship between data	No or limited control over proprietary data formats; systematic and consistent metadata collection beyond instrument metadata can require changes in workflows and instrument management	FAIR data; increased research impact; best practices in data management; standardisation of metadata for data annotation and description
Variability	Number of inconsistencies in the data (e.g. outliers), or inconsistent rate at which data are created or stored	Identification of inconsistent data prior to data processing and analysis	Inconsistent data (anomaly, outlier) might be a signature of a real property of the sample examined (e.g. rare events, weak interaction)
Volatility	Lifetime of data; how long data should be stored for or kept accessible	Maintaining storage and accessibility while data are kept	Optimisation of data storage through the implementation of data retention and disposal policies

## Big-data electron microscopy at research facilities

The advent of big data in EM has certainly impacted researchers. They are often overwhelmed by the vast amount of digital information collected in a single acquisition session. Improved highly sensitive cameras, collection of large volumes of data and high-throughput collection of numerous single-image planes are a few of examples attributed to this big-data challenge. In parallel, research facilities, institutes and centres that host big-data-producing electron microscopes have accompanied this revolution to maintain their services to their research communities. Given the high level of expertise and the associated costs for the operation and maintenance of these advanced microscopes, EM as a high-end imaging and analytical tool is mostly available at core facilities or dedicated centres at the institutional level that support research communities rather than a single research group. Facilities design or co-design workflows and practical approaches associated with microscopes (e.g. for data storage, transfer, processing and quality control), and are often involved in the development of the software and hardware infrastructure underpinning those workflows. Furthermore, they also provide expert advice on a range of commercially available software solutions (e.g. for data analysis and visualisation), promote best practices in research data management and offer training for instrument scientists and researchers at varied levels (Braet and Ratinac 2007; Alewijnse et al. 2017; Mills 2021). Facilities contribute to standardising workflows, fostering innovations and improving EM accessibility by both lowering the barrier to entry for non-expert users and optimising workflows to maximise the availability of instruments (Zimanyi et al. 2022). They also keep researchers at the forefront of technological development. Research infrastructure facilities – preferably through a centralised and coordinated approach – therefore play a critical role in the overall performance of microscopes, that is, in their reliability, efficient and optimal use and quality output.

### The big-data revolution at microscopy research facilities

A recent report by Poger et al. investigated how microscopy research facilities in Australia, France, Germany, the Netherlands and the USA adapted to the big-data revolution in an environment where research data were increasingly required to follow the FAIR principles (Poger et al. 2021). Seventeen facilities in total were interviewed. All facilities were either located at universities or primarily supporting academic researchers. The microscopy techniques cited as creating or likely to create problems included scanning EM (SEM), transmission EM (TEM), scanning transmission EM (STEM), focused ion beam systems (FIB) and derived techniques such as cryogenic EM (cryo-EM), focused ion beam–scanning EM (FIB-SEM) and correlative light and EM (CLEM). Some facilities indicated that a single cryo-TEM experiment could generate in excess of 1–2 TB of data per day. The total volume of data generated at some facilities amounted to between 500 TB and 2 PB per year. The report reviewed workflows, tools, methods, procedures and the whole underlying infrastructure used for data transfer, storage and overall data management, as well as data processing. It was found that, in general, the challenges posed by the vast amount of data and the trends to adapt to them were shared across all facilities, regardless of their geographical locations, sizes and levels of specialisation. Note, some facilities operated a diverse range of instruments and supported various research fields, whereas other facilities were specialised in biological cryo-EM. In general, all facilities indicated that data processing had shifted towards high-end workstations or high-performance computing in order to deal with the high volume and velocity of data.

While the above-mentioned report mostly highlighted issues associated with big data, it demonstrated that the challenges of big data in EM could be tackled by harnessing suitable tools and adopting appropriate methods and practices to transfer, analyse and manage data. Some of them are described in this review. The report showed that researchers and facilities needed to integrate approaches developed in e-science (alternatively called e-research in Australasia and cyberinfrastructure in the USA) to ensure the sustainability of big-data EM and the promotion and adoption of the FAIR principles across facilities and researchers. Some of the findings and conclusions on data transfer and management are outlined below.

### Data transfer at microscopy research facilities

**Fig. 2:**
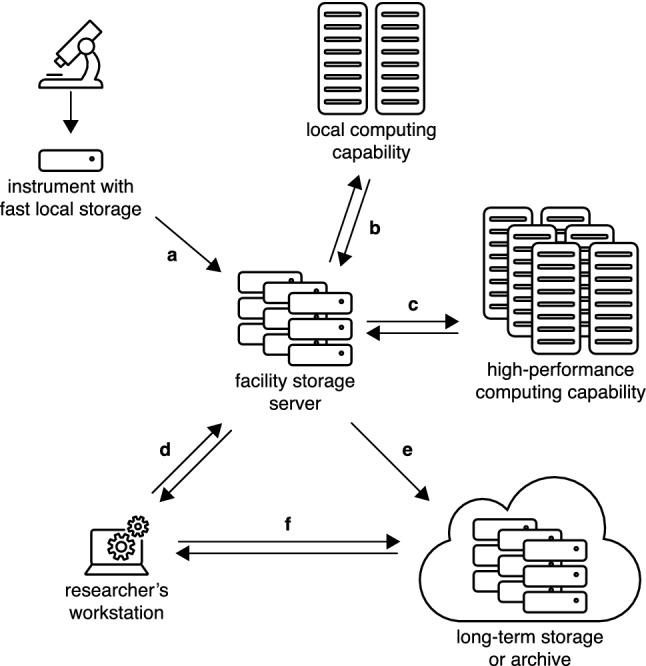
Schematic representation of the different workflows involved in data transfer at a microscopy research facility. **a** From instrument to facility data storage; **b** and **c** from facility storage to computing capabilities; **d** from facility storage to researchers’ individual workstations; **e** from facility storage to long-term storage or archive; **f** from researchers’ workstations to long-term storage or archive

Data transfer was a typical illustration of an area that had been impacted by the big-data revolution, mostly by the creation of large volumes of data at high rates (Poger et al. 2021). The report focused on the transfer of data between storage servers at facilities and various other end points: instruments, remote and local computing capabilities, end users’ computers, institutional repositories and partner institutions (Fig. 2). Data transfer often consisted of routine tasks that required human intervention for actuation or ad hoc adjustments. Sometimes, data transfer was via “sneakernet”, that is, using flash drives or emails. The tools or settings used for transfer could result in bottlenecks in data workflows that limited the capacity to process large data volumes or high-velocity data following acquisition. In particular, some facilities cited limitations in their capacity to process or pre-process data on-the-fly, i.e. in real time or near-real time as data were generated from instruments. This was described as especially important for on-the-fly monitoring of data quality as it ensured that the time dedicated to data collection using instruments at facilities led to high-quality data. Optimal microscope usage was therefore coupled to appropriate workflows to move data. Efficient data transfer was seen as necessary to automate data collection and to develop high-throughput EM. The report noted limited knowledge of, or awareness of, network performance across the majority of the facilities interviewed. This impacted the time taken to transfer data, the reliability, reproducibility and predictability of the tools chosen to transfer data, and the ability to optimise and automate workflows.

### Data management at microscopy research facilities

Research data management, especially data storage (retention and disposal) and description (metadata collection), was an emerging challenge in the context of big data and compliance with the FAIR principles. In general, most facilities showed some level of understanding of data management. Best practices in research data management were considered important to ensure that the quality and the integrity of research data were maintained throughout the data lifecycle (collection, organisation, storage, preservation and sharing) and that legal, regulatory, ethical, governance and funding requirements were met. All the facilities applied some level of local data management (in particular for data storage), but general guidelines, especially on data retention and disposal, were needed given the increasing volumes of data generated by instruments. Many facilities stated that they were using, had used, or had trialled a number of tools to assist them in data and image management, including OMERO (Allan et al. 2012; Burel et al. 2015; Li et al. 2016), XNAT (Marcus et al. 2007), 4CeeD (Nguyen et al. 2017) and MyTardis (Androulakis et al. 2008; Meyer et al. 2014). While all facilities acknowledged how useful those tools could be, in particular in the standardisation of workflows and the promotion of the FAIR principles through the collection of metadata, a large number of facilities that operated a broad and diverse range of instruments indicated that finding a solution that would be suitable for all or most instruments was in fact difficult. For example, the file formats supported by OMERO was limited to those found in life sciences. Facilities often indicated that proprietary formats were converted to standard, open-source file formats, so data interoperability was in general not cited as a major hurdle for data management and FAIR data. The number of file formats used at a facility could however be challenging, especially at multidisciplinary facilities (some facilities managed over 50 formats). Most facilities commented that data-processing and data-analysis tools also led to a multiplication of file formats. Metadata collection at all the facilities concerned instrument metadata only, that is, metadata generated by the instrument alongside the data (embedded in data files or in separate files). In contrast, metadata that included general information to describe data (such as title of the dataset and names of the researchers involved) and that are essential to enrich data to a higher FAIR state were never collected. The nature of instrument metadata and the range of metadata captured and stored could vary across instruments, acquisition software and file formats within a facility and between facilities. Some facilities noted that many proprietary formats contained some form of metadata embedded (such as energies and scan time) but the conversion to standard or open-source formats (e.g. TIFF and BMP) often resulted in the loss of those metadata.

In general, whether it was for data transfer or management, an increased international awareness and call to standardise the way that data were handled at microscopy research facilities were noted, with the ultimate goal that data were accessible to all for scientific purposes and reproducibility.

## Transfer of big data

Efficient and sustainable data transfer lies at the core of the operations of microscopy facilities. Data transfer is a critical component in the automation of data collection and processing workflows and overall data orchestration through to long-term storage, as well as in the development of high-throughput EM (Suloway et al. 2005; Scherer et al. 2014; Ding et al. 2015; Cheng et al. 2021). The efficiency and sustainability of data transfer rest upon three fundamental properties of the network infrastructure: reliability, predictability and speed. In the case of big data, uncertainties and shortcomings in these three properties have only become more acute. Inconsistent transfer of big data over a complex network infrastructure may reveal existing weaknesses in components of the infrastructure (hardware, software or workflows) that were either hidden or manageable before the big-data revolution. Fortunately, a range of tools are available to ensure fast, reliable and predictable data transfer. They are introduced below and listed in Table 2.

**Table 2:** Tools that can assist in dealing with challenges posed by big data in cryo-electron microscopy (cryo-EM) and tomography (cryo-ET)

Big-data challenge	Tool	References	Comment
Data transfer			
File-transfer service	Globus	www.globus.org (Foster 2011; Allen et al. 2012)	
	rclone	rclone.org	Useful for workflow optimisation
Network performance (baselining and benchmarking)	perfSONAR	www.perfsonar.net (Hanemann et al. 2005)	
	RIPE Atlas	atlas.ripe.net	
	SolarWinds	www.solarwinds.com	Commercial solution
Data processing and analysis			
Virtual research environments (VREs)	ScipionCloud	(Cuenca-Alba et al. 2017)	
	COSMIC^2^	(Cianfrocco et al. 2017)	Available in the USA
	Electron Microscopy Data-Processing Portal	(van Schyndel 2022)	Available in Australia
	Characterisation Virtual Laboratory	imagingtools.au/characterisation-virtual-laboratory	Available in Australia
	Virtual Desktop Service	desktop.rc.nectar.org.au	Available in Australia
	Australian Research Environment	nci.org.au/our-services/data-services	Available in Australia
Cloud-computing workflow	cryoem-cloud-tools	(Cianfrocco et al. 2018)	Uses Amazon Web Services
	ZeroCostDL4Mic	(von Chamier et al. 2021)	Uses Google Colab to run deep-learning-based programs (e.g. image segmentation, object detection and denoising)
Artificial-intelligence-based solutions	DeepPicker	(Wang et al. 2016)	Particle picking (cryo-EM)
	DeepFinder	(Moebel et al. 2021)	Identification of macromolecules (cryo-ET)
	Topaz-Denoise	(Bepler et al. 2020)	Image denoising
	AD_LTEM	(Zhou et al. 2021)	Enhanced resolution and sensitivity
	DoG Picker	(Voss et al. 2009)	Particle picking (cryo-EM)
	TiltPicker	(Voss et al. 2009)	Particle picking from image tilt pairs (cryo-EM)
	DeepEMhancer	(Sanchez-Garcia et al. 2021)	Post-processing of cryo-EM maps
Data management			
Image data management	OMERO	(Allan et al. 2012; Burel et al. 2015; Li et al. 2016)	
	Pitschi	(Nguyen 2022)	
	NexusLIMS	(Taillon et al. 2021)	
	XNAT	(Marcus et al. 2007)	
	MyTardis	(Androulakis et al. 2008; Meyer et al. 2014)	
User training	MyScope	myscope.training	

### Achieving fast data transfer

The high volume, variety or velocity of data collected requires rapid transfer to data-processing computers and data stores that can achieve at least 10 Gb/s transfer speed. Hence, fibre-optic cables are preferred over copper-based connections because they provide more bandwidth than copper cables of the same diameter (Sader et al. 2020; Mills 2021). Traditional protocols and tools for data transfer such as SFTP, SCP, Robocopy, rclone and rsync are widely used but have limitations when it comes to achieving high-speed data throughput on fast networks (10 Gb/s and above). These tools are prone to failure or reduced efficiency in cases of high network latency, loss or congestion. Network latency is when a delay is observed to transfer data between two end points. Several factors contribute to network latency, including the distance between the two end points (that is, the longer the distance, the higher the latency), and the nature and configuration of the components of the network infrastructure (e.g. security measures). To overcome such limitations and to standardise data transfer across operating systems, higher-level tools are required. Amongst those tools is Globus (www.globus.org), a service developed for researchers and research organisations that provides fast, reliable, secure and high-assurance transfer of data (Foster 2011; Allen et al. 2012). Unlike tools such as rsync, SCP and SFTP, Globus is less sensitive to network-performance fluctuations (e.g. glitches and high latency) and checks the integrity and completeness of data transfer. It can be used to transfer data between a range of storage devices and systems, such as personal computers, data stores, compute servers (e.g. high-performance computing facilities) and cloud stores (including commercial solutions such as DropBox, Amazon Web Services, Microsoft OneDrive, Microsoft Azure, IBM Cloud, Google Cloud and Google Drive). Globus was recently tested across several university-based microscopy core facilities in Australia (van Schyndel et al. 2021). It was shown to be especially suitable to transfer large data volumes across long and short distances, which helped streamline workflows for data collection, processing and analysis.

### Monitoring network performance

In a typical workflow that includes storage, processing and analysis of data, data are transferred multiple times as illustrated in Fig. 2:

- from instrument to facility data storage (transfer denoted as a in Fig. 2): on-instrument storage is prioritised for speed so that data can be captured quickly during measurements. However, the data need to be transferred regularly to larger, short- or mid-term storage because on-instrument storage can fill up within hours;
- from facility storage to computing capabilities (transfers b and c): data are transferred to a local, in-house computing capability at the microscopy facility (a computer, a local computing server or a virtual desktop) (b) or to an external high-performance computing (HPC) capability that may be located in the same institution as the microscopy facility or outside (c). While near-real-time or real-time data analysis may be performed to assess data quality and optimise instrument use, in-depth data processing and analysis often necessitates appropriate HPC resources;
- from facility storage to researchers’ individual workstations (transfer d): researchers may choose to store a copy of their data on their computers. Data processing and analysis require high-end workstations provided datasets are not too large;
- from facility storage or researchers’ workstations to long-term storage or archive (transfers e and f): it is not practical nor possible to store the growing data volumes generated by instruments on the facility storage servers or on researchers’ computers. Once data have been processed and analysed and regular access to data is no longer required; the data can be kept in an appropriate and sustainable form of long-term storage or archived, which can be local, remote or in the cloud. Note, data can be retrieved from the long-term storage or archive.

**Fig. 3:**
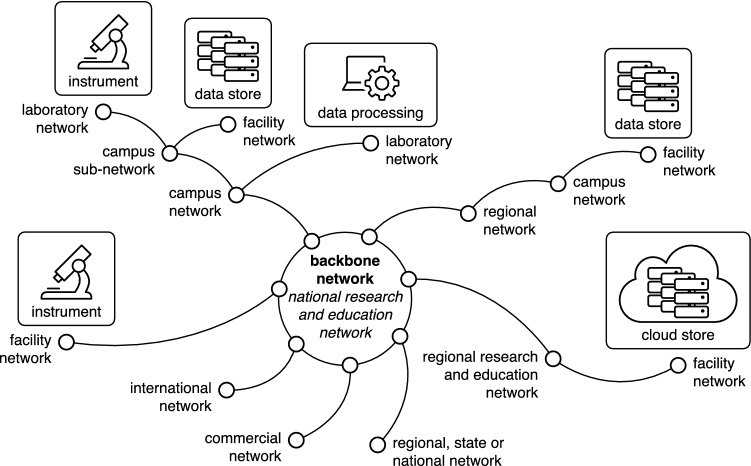
Schematic representation of a typical multi-component network composed of interlinked national, international, institutional or local networks or subnetworks

Figure 2 shows multiple instances of data transfer between end points that may be geographically close or distant. However, it hides the complexity of the underpinning global network infrastructure that crosses the boundaries of facilities, organisations and smaller network components. Behind the apparent simplicity of the task from a user’s perspective, transferring microscopy data from one point to another implies that data may transit via a series of nodes along a network path. Indeed, the schematic in Fig. 3 illustrates that computer networks at universities and other research organisations, and across cities and countries, are complex, multi-component systems sometimes consisting of subnetworks. For example, a network at a university campus may comprise subnetworks that vary in technical specifications such as the types of cables used (for example, fibre-optic cables and copper cables). Note, Fig. 3 represents an example of how end points may be interconnected. In some countries, there may be no regional network or no regional research and education network. A common feature across all countries is the presence of a national research and education network (NREN). NRENs are specialised internet service providers that support the needs of their national research and education communities through a high-speed backbone network (for example, AARNet in Australia, Internet2 and ESnet in the USA, Jisc in the UK and RENATER in France). Access of data-intensive facilities such as microscopy research facilities and data-processing and storage facilities to NREN infrastructure is vital to maintain flows of large data volumes between geographically dispersed points at high speed, which are especially required in near-real-time or real-time applications. This is permitted by the inherent nature of the backbone networks provided by NRENs which are, by design, over-dimensioned with respect to the average network usage. This large amount of white space enables NREN networks to deal with spikes of high demand while minimising loss and congestion during data transfer. This is in contrast with commercial networks that aim to keep white space low, i.e. to minimise unused network capacity.

However fast data transfer may be, a reliable and predictable network infrastructure is essential. Instabilities, discrepancies or faults in the behaviour of interconnected networks can have various potential sources. Identifying problems and recording when they happen in such an environment is a challenge for research organisations and NRENs. Network reliability and predictability are measured through network performance, which is itself a composite property consisting of qualitative and quantitative properties that collectively characterise the end user’s experience of the service supported by a network at a point in time or over a period of time (Myers and Poger 2022). Network baselining is monitoring and measuring the performance of a network over time. Benchmarking the performance of a network is comparing its performance with respect to that of another network, an industry standard or any other external reference. Benchmarking determines whether a network behaves normally (within a range of acceptable parameter values) or as expected. Network baselining and benchmarking are essential to monitor and evaluate the reliability and predictability of the performance of a network. They can be used by NRENs, IT specialists at universities and research organisations, and research facilities and researchers to set expectations regarding the capacity for a network to transfer data. Importantly, network baselining is an iterative process: whenever a component of a network infrastructure changes (e.g. hardware, software, software version in any part of the network from end to end), it is necessary to repeat network performance baselining to compare with the previous performance baseline and ensure that the quality of the service supported by the network is maintained. A range of tools are available to observe, record, understand and predict network performance between end points (e.g. instruments, data stores and compute facilities) within an organisation or between different organisations. Those tools include free programs such as perfSONAR (www.perfsonar.net) and RIPE Atlas (atlas.ripe.net), and commercial solutions such as SolarWinds (www.solarwinds.com). The perfSONAR tool, for example, is a dedicated open-source, modular toolkit for network performance monitoring to support research and education (Hanemann et al. 2005). It contains a suite of tools and services (e.g. ping, iperf3, traceroute) that measure the capacity and the quality of a network as well as the consistency of network behaviour in real time across an entire end-to-end network path. Measurements can be recorded over time for retrospective analysis. It detects and diagnoses issues or anomalies, and facilitates the collection and sharing of network performance information. This makes perfSONAR very well suited to network baselining and benchmarking. It allows one to predict the performance of a network and determines if a network has the capacity to meet users’ expectations regarding the reliability for data transfer. This is of great importance for large volumes of high-velocity data. perfSONAR is therefore useful to end users and microscopy facilities that rely heavily upon well-performing networks for big-data transfer within and between institutions (Myers and Poger 2022).

## Processing and analysis of big data

A single EM experiment can now amount to terabytes of data. Two major factors have contributed to this. First, advances in direct electron detection have led to an increase in the amount of data collected in the form of high-resolution movies of up to 100 frames per imaging area (Baldwin et al. 2018). Secondly, approaches to automate pipelines for data collection and processing have been developed, for example with Leginon (Suloway et al. 2005; Cheng et al. 2021), SerialEM (Mastronarde 2005), Appion (Lander et al. 2009), Scipion (de la Rosa-Trevín et al. 2016), Focus (Biyani et al. 2017), CryoFLARE (Schenk et al. 2020) and the Caltech Tomography Database and automatic image processing pipeline (Ding et al. 2015). Such progress in how EM data are processed and analysed has been underpinned by changes in techniques, technologies and infrastructure, in particular in the area of artificial intelligence (AI), computing and workflow automation.

### EM big data and artificial intelligence

AI using machine- or deep-learning methods is increasingly being used in data processing and analysis to extract meaningful information from large datasets. Examples include improving the resolution and sensitivity of electron microscopes (Zhou et al. 2021), reducing noise in datasets (Bepler et al. 2020) and facilitating and automating pattern recognition such as particle selection (Voss et al. 2009; Wang et al. 2016; Sanchez-Garcia et al. 2021), macromolecule identification (Moebel et al. 2021; Uddin et al. 2021; Che et al. 2018) and cell counting and classification (Liu et al. 2019; Zaritsky et al. 2021) (Table 2). Of particular interest is the European-Union-funded project AI4LIFE (https://ai4life.eurobioimaging.eu) that started in 2022 and aims to develop and make readily accessible methods based on AI for bioimage analysis, in particular microscopy image analysis. Note, it is commonly believed that algorithms can be trained better with more data and consequently provide more accurate results. However, this is not necessarily true nor possible with big data. In particular, several traditional machine-learning algorithms were designed for smaller datasets assuming that entire datasets could be stored in memory or that they were available for processing at the time of training, which is often impossible with big data (L’Heureux et al. 2017).

### Computing EM big data

Advances in high-performance computing (HPC) using both central processing units (CPUs) and graphics processing units (GPUs) concomitant with the development of better algorithms, particularly those amenable to parallelisation have been critical to harness big data (Baldwin et al. 2018). They have been especially exploited in a range of tools that have helped researchers leverage the big-data revolution in cryogenic electron microscopy and tomography. Such applications include RELION (Scheres 2012; Kimanius et al. 2021), cryoSPARC (Punjani et al. 2017), *cis*TEM (Grant et al. 2018), EMAN2 (Chen et al. 2019) and emClarity (Himes and Zhang 2018). Some of these are continually updated to facilitate and accelerate data processing and analysis. For example, RELION, which is widely used in cryo-EM and cryo-ET, has been regularly updated to integrate new algorithms and optimisations to improve efficiency and expand its range of functionalities (Scheres 2012; Bharat et al. 2015; Scheres 2015; Scheres 2016; He and Scheres 2017; Zivanov et al. 2020). In addition, a pipeline approach of RELION based on standardised and (semi-)automated procedures for structure determination has been developed to allow on-the-fly processing of cryo-EM data (Fernandez-Leiro and Scheres 2017; Kimanius et al. 2021). Finally, RELION supports GPU acceleration (Kimanius et al. 2016) and CPU vector acceleration (Zivanov et al. 2018) to reduce the computational load. Although programs such as RELION, cryoSPARC and *cis*TEM tend to scale well to larger resources thanks to CPU or GPU parallelisation, they still present high computational costs for researchers, especially for large datasets which require high-end workstations or computer clusters. Therefore, the bottleneck in big-data processing and analysis does not generally lie in the unsuitability or limited scalability of existing software to deal with big data, but in the underlying compute resources and workflows.

Large computational resources (high-end workstations or computer clusters such as high-performance computers) may be too expensive, inaccessible or unavailable to many laboratories and researchers. In addition, HPC capabilities need to be managed and regularly expanded to meet growing compute and storage demands. Cloud computing using commercial solutions such as Amazon Web Services (AWS), Microsoft Azure and Google Cloud Platform, have been suggested as viable, flexible and cost-effective alternatives to traditional, academic computing environments (Cianfrocco and Leschziner 2015; Castaño-Díez 2017; Cuenca-Alba et al. 2017). The limited command-line literacy of new EM users or novice researchers can be another major barrier to big-data EM because they require training to use a Linux/Unix environment and deal with a HPC environment for at least job submission and management. To address this obstacle, user-friendly alternatives with graphical user interfaces (GUIs) as well as integrated and standardised workflows have been developed. GUI-based solutions have gained in popularity as they enable any user to exploit the power of a HPC infrastructure (supercomputers, cloud) for data processing and analysis in a simple and flexible way through a web-based virtual desktop that can be accessed from anywhere. They are called various names: virtual desktop infrastructure, virtual computing environments, science gateways (mainly in the USA), virtual research environments (mainly in Europe), virtual laboratories (mainly in Australia), “collaboratory” (Wulf 1993) or, more generically, research platforms, portals or workbenches amongst other denominations. Herein, we refer to these collectively as virtual research environments (VREs). In general, VREs provide convenient and secure access to CPU or GPU computing at no cost or at a limited cost (such as a pay-as-you-go basis), thereby avoiding investments in hardware and long-term maintenance for research laboratories. For research organisations, they are cost-effective as their flexibility and versatility allow them to serve diverse user communities. Examples of VREs for EM data processing include ScipionCloud (Cuenca-Alba et al. 2017), COSMIC^2^ in the USA (Cianfrocco et al. 2017) and, in Australia, the Electron Microscopy Data-Processing Portal (van Schyndel 2022), the Characterisation Virtual Laboratory (imagingtools.au/characterisation-virtual-laboratory), the Virtual Desktop Service (desktop.rc.nectar.org.au) and the Australian Research Environment (nci.org.au/our-services/data-services) (Table 2). While some VREs such as ScipionCloud are agnostic in terms of the underpinning computing infrastructure, others have been developed to be deployed on commercial, institutional or national infrastructure, which may restrict their accessibility and the general awareness of their existence or availability in the research community. For example, the Electron Microscopy Data-Processing Portal and the Australian Research Environment use national research resources funded by the Australian government (the ARDC Nectar Research Cloud and the National Computational Infrastructure, respectively). This means that they are available to the Australian research community only. Regardless of their technical details, successful and impactful VREs, however, require adequate ongoing support (e.g. funding and skilled professionals) to ensure long-term sustainability, significant community adoption, persistent online presence, technological relevance and compatibility with advances in technologies and standards in computing, data management and cybersecurity (Calyam et al. 2021). Importantly, the high degree of user-friendliness of VREs should not be at the expense of the reproducibility of the results and repeatability of the tasks that they facilitate. While VREs can lower the entry barrier to advanced CPU- or GPU-based data processing and analysis methods, critical information on the programs or automated pipelines available in VREs should be easily accessible to users. For example, the versions of the programs used, which options were turned on or off during software installation, the nature of the intermediate steps executed between the initial (input) and final (output) data, the treatment of outliers and missing values and the full set of model parameters used during processing or analysis are crucial factors that determine the reproducibility and repeatability of an experiment (Baker 2016; Stodden et al. 2016; Taubert and Bucker 2017). Funding bodies and research-infrastructure organisations have encouraged and enabled the development and deployment of VREs in multiple disciplines. To ensure long-term viability, there is clearly a need for consolidation, interoperability and a better coordinated approach in the development and deployment of VREs, including in the establishment of common policies, governance strategies and best practices (for example, on underlying architectures, interfaces, data access and community building). At the international level, the Research Data Alliance has thus established the VRE Interest Group (rd-alliance.org/groups/vre-ig.html) to explore those challenges and to provide expert ongoing guidance in this ever-changing EM computing world.

Alongside VREs are workflows that integrate cloud computing seamlessly for users. For example, cryoem-cloud-tools moves cryo-EM analysis routines and atomic-model-building jobs in the RELION processing pipeline to Amazon Web Services and synchronises data in real time between the cloud and the user’s computer (Cianfrocco et al. 2018). In the area of data analysis, the platform ZeroCostDL4Mic is an entry-level tool that simplifies the use of deep learning in microscopy image analysis. It integrates cloud-based virtual machines provided by Google Colab (von Chamier et al. 2021) (Table 2).

### Workflow optimisation

Optimisation of workflows is critical and can have a significant effect in the ability to process big data. Importantly, optimising some tasks in a workflow can be readily achievable (Silver 2022). For example, default and legacy settings in computers and network tools as well as legacy or traditional tools or scripts used for data movement may be inadequate for big data. These tools, scripts and settings may have been used for many years (sometimes decades) at a microscopy facility and are therefore well established and familiar to facility support staff and their research users. Moving to newer and more modern approaches may offer more advanced settings, be better suited to recent standards and infrastructure, and have a more user-friendly GUI (instead of command lines). Facilities thus require resources to investigate workflow improvements, implement the changes and provide the training and support to their research communities. This underscores how critical collaborations between microscopy research facilities and IT providers or e-science specialists are to ensure that research facilities and their support staff can exploit technological advances. Importantly, the adaptation of workflows to big data should not be reduced to investment in new hardware.

Data transfer from the instrument computer to temporary storage or from temporary storage to computing capabilities (transfers a, b and c in Fig. 2) can be a bottleneck for big data because of the high volumes, velocity or variety of the data collected. Various tools such as SCP, SFTP, rsync and rclone have been commonly used combined with task schedulers (for example, cron, Windows Task Scheduler and systemd) in (semi-)automated data-movement workflows. However, SCP, SFTP and rsync have intrinsic limitations that hinder the high throughput required by big-data EM. Briefly, rsync does not support parallel data-transfer streams and, as a dial-up-age tool, performs poorly on today’s multi-gigagbit-per-second connections. Despite their higher transfer speed due to concurrent transfer streams, SFTP and SCP have throughput limitations because of their encryption algorithms. In contrast, rclone is a more modern program that can sustain elevated transfer rates over 1 Gb/s. However, it is important to note that, ultimately, transfer rates are by nature limited by the read-and-write speed of disks (referred to as I/O speed) and network bandwidths. A key feature of rclone is that it supports many common protocols and application programming interfaces (APIs) to transfers to and from a range of locations, including cloud storage. The underlying model for data transfer is another important factor: data can be taken out of the instrument computer by the storage server (pulling) or put into storage by the instrument computer (pushing). The CPU load is on the instrument computer in the latter case, whereas it is on the storage server in the former. Switching from a push to a pull may lead to a great increase in data-transfer rate and reduced time to transfer data. It frees up computing power in the instrument computer that can be solely dedicated to capture images and perform other tasks on images, such as the conversion to other formats. For example, converting MRC images to a TIFF format is CPU-intensive. In an optimised big-data workflow, using the TIFF format with lossless compression (LZW and ZIP methods) is especially advantageous as it is a lossless image compression that provides storage-space saving by a factor of five on average (Eng et al. 2019). This also translates into faster transfer times as datasets are smaller. Overall, such apparently small changes to optimise an EM workflow and harness big data can produce dramatic changes, such as enabling on-the-fly data processing for real-time or near-real-time data-quality assessment and optimised instrument usage (Silver 2022).

## Management of big data

Data management is the cardinal activity that underpins the value of EM big data. Amongst the ten V’s of EM big data (see Section “Big data in electron microscopy” and Table 1), value is closely associated to veracity, visibility, vocabulary and volatility, which all depend on the adoption of best practices in data management by research facilities and researchers. The management of EM big data faces three concurrent challenges: (1) the need for community-wide standards and conventions for data description, annotation, storage and sharing; (2) the capacity to handle the data deluge; and (3) the operationalisation of the FAIR and CARE principles. User training and community uptake of standards, conventions and best practices are paramount to ensure that EM big data are managed in a way that is appropriate and sustainable. In particular, managing big data may require a change in practices and an understanding of the overall workflows to manage the big-data deluge efficiently (Sader et al. 2020; Alewijnse et al. 2017). Online learning environments such as MyScope developed by Microscopy Australia (myscope.training) and its module on research data management facilitate user training and the early adoption of best practices to support the FAIR principles.

### Establishing standards and conventions

Big data have created new opportunities to the EM community. Data variety and data vocabulary are key attributes in the establishment of standards and convention for EM data. The various proprietary and non-proprietary data formats created at the time of data capture, processing and analysis often lead to the creation of files that contain non-standardised metadata, incomplete metadata or no metadata at all for data annotation or description. Combined with the lack of workflows that allow for the systematic and consistent collection of standardised generic metadata, there is a significant risk that the value of EM big data may be low. In addition, multidisciplinary microscopy research facilities can manage over 50 different file formats, which is not sustainable in the long term (Poger et al. 2021). It is therefore essential to develop, promote and adopt rigorous international standards for annotating, describing and formatting microscope image data beyond legitimate reasons that software developers may have for using proprietary data formats or non-conventional metadata. However, it is not sufficient to define standards and conventions. For those to be adopted, it is pivotal that the science community be provided with the necessary software libraries to allow lossless data conversion to and from the convention or standard (Patwardhan et al. 2012). Bio-Formats is an excellent example of such a library that aims to promote the open standard called the OME (Open Microscopy Environment) data model (Goldberg et al. 2005; Linkert et al. 2010).

Standards facilitate reproducibility of experiments and data reuse, encourage research transparency and data sharing, and contribute to the development of interoperable ecosystems of tools for processing, analysis and visualisation of data. Repositories are powerful tools to standardise data and metadata and allow seamless data sharing. The EM field has been a forerunner in setting up public microscopy repositories for EM data. Good examples are the Electron Microscopy Data Bank for 3D-structure data (EMDB or EMDataBank) (Lawson et al. 2015) and the Electron Microscopy Public Image Archive (EMPIAR), a public archive for raw 2D image data that also supports 3D-structure data deposited in EMDB (Iudin et al. 2016). Noteworthily, the Protein Data Bank (PDB) stores atomic models constructed using EMDB data (Berman et al. 2000, 2003). More broadly, and for completeness, the repositories BioImage Archive for published image data (Hartley et al. 2022) and Image Data Resource (IDR) for reference data with added value (Williams et al. 2017) are available to all data irrespective of the imaging technique utilised.

There are many initiatives currently that guide the field of light microscopy towards standardisation that may benefit EM. For example, one of the goals of the AI4LIFE project is to develop standards by creating harmonised and interoperable tools and methods, in particular in the submission, storage and FAIR access of reference data, reference annotations and AI methods (https://ai4life.eurobioimaging.eu/about-us/#objective). Recently, the Recommended Metadata for Biological Images (REMBI) were proposed as metadata guidelines for light and electron microscopy (Sarkans et al. 2021). Other notable efforts include the development of guidelines for Minimum Information about Highly Multiplexed Tissue Imaging (MITI) for data and metadata in genomics and microscopy of tissue images (Schapiro et al. 2022), alongside the establishment of the 3D Microscopy Metadata Standards (3D-MMS) by the Brain Research through Advancing Innovative Neurotechnologies (BRAIN) Initiative and the wider neuroscience research community (Ropelewski et al. 2022). Similarly, the Brain Imaging Data Structure (BIDS), a standard initially developed for neuroimaging data and metadata for magnetic resonance imaging (Gorgolewski et al. 2016), has been extended to microscopy (Microscopy-BIDS) to support common imaging methods, including optical and electron microscopy with the aim to harmonise metadata definitions for hardware, image acquisition and sample properties in multi-modal, multi-scale imaging (Bourget et al. 2022). The initiative QUality Assessment and REProducibility for instruments and images in Light Microscopy (QUAREP-LiMi) plans to improve data quality and experiments reproducibility through the development of common standards, guidelines, metadata models and tools (Boehm et al. 2021; Nelson et al. 2021). Amongst the achievements of QUAREP-LiMi are the 4DN-BINA-OME (NBO) Microscopy Metadata specifications framework (Hammer et al. 2021) and three interoperable metadata collection tools, namely Micro-Meta App (Rigano et al. 2021), MethodsJ2 (Ryan et al. 2021) and MDEmic (Kunis et al. 2021). Importantly, many of these initiatives build on existing standards and tools to maximise sustainability, interoperability and adoption by the community. Specifically, the tools MethodsJ2 is an ImageJ/Fiji plugin (Schneider et al. 2012; Schindelin et al. 2012; Rueden et al. 2017); MDEmic is fully compatible with Bio-Formats and the OME data model, and is part of the standard installation package of the image database OMERO (under the name OMERO.mde). MethodsJ2, MDEmic and Micro-Meta App also interoperate with each other so they can create a rich environment conducive to metadata collection. Regarding standards, 4DN-BINA-OME and 3D-MMS are extensions of existing metadata standards (the OME data model in both cases, as well as the generic DataCite metadata schema for 3D-MMS).

Global community-driven partnerships such as the pan-European consortium Euro-BioImaging (eurobioimaging.eu), BioImaging North America (bioimagingnorthamerica.org) and Global BioImaging (globalbioimaging.org) in conjunction with national networks play an important role in the cooperative development and dissemination of best practices, standards and conventions for formats, repositories, annotation, description, processing, visualisation and analysis of image data beyond geographical boundaries, techniques and disciplines (Swedlow et al. 2021). In Australia, Microscopy Australia promotes and coordinates the adoption of guidelines for the collection of metadata based on the DataCite schema (schema.datacite.org), across material sciences and life sciences and for all microscopy modalities. In particular, the guidelines being developed for metadata collection require a set of minimum metadata properties being collected and described using consistent information.

### Managing the big-data deluge

In principle, the management of EM big data is not fundamentally different from that of “small data”. However, specific attributes of EM big data may reveal existing flaws or issues in infrastructure (hardware, software or workflow) by stretching it to its limits. In particular, data storage, manual operations and methods to read and write smaller files or datasets that have so far been suitable may not be compatible nor practical with large datasets.

An important consequence of the data deluge is the long-term storage, archiving and preservation of microscopy data (and their accompanying metadata if they are stored in separate files). This all is associated with data volatility (Table 1). Storing data is the first step in data management. Big data require highly scalable storage systems at a reasonable cost. As the volumes of data generated by single experiments can amount to terabytes in EM nowadays, it is essential that scientists and research organisations assess how important each dataset is and more importantly, understand how long each dataset should be kept for. Research organisations are expected by law or funders’ requirement to retain categories of research data for specific retention periods before disposing of them. However, datasets often exist in multiple copies across different storage systems or under different user accounts within an organisation that are never or rarely destroyed. This is not a sustainable practice for evident reasons. In addition, there must be minimum information associated with the stored data for them to keep their value over their lifetimes. That implies organisational or, preferably, community-endorsed universal standards and conventions to establish minimum information requirements. This includes the types of metadata to collect, the data formats to keep and specific directives on minimal storage time. Finally, provisions should be taken for long-term storage and backup of original data files. For the latter, chief investigators have a central role to play to foster best practices and ensure research data integrity.

The use of AI to assist in particle picking in cryo-EM and macromolecule picking in cryo-ET is an efficient and time-effective avenue to automate complex, laborious, tedious and time-consuming tasks when completed manually by an expert. When trained properly, deep-learning methods can provide fast and reliable results on large datasets (Wang et al. 2016; Moebel et al. 2021). Furthermore, automation promotes consistent and standardised image annotation using, for example, controlled terms, which in turn can contribute to the adoption of common machine-readable metadata standards and facilitate interoperability between the various programs used in processing and analysis pipelines that use annotation metadata.

Beyond the standardisation of data formats, the deluge of massive volumes of data creates bottlenecks that cannot be solved by new hardware, workflow optimisation or automation of tasks. For files greater than 10 GB in size, the repeated access to large datasets in proprietary file formats for on-the-fly translation or permanent conversion into open formats such as OME-TIFF and HDF5 can come at such a great computational cost or take so much time that it precludes the use of data- and resource-hungry applications such as training of artificial intelligence models and visualisation in public repositories (Moore et al. 2021a). This is because the representation of data in traditional image formats such as TIFF requires that the whole image data be loaded into computer memory when the file is opened or displayed on a screen. This becomes impractical or impossible when the size of an image exceeds that of a computer memory. Traditional image formats are consequently ill-suited for repeated, frequent access to data. In contrast, pyramidal images use a multi-resolution representation of data that enables zoomable visualisation and selectable levels of resolution for interactive navigation and scalable image analysis (Moore et al. 2021a, 2021b). The image is composed of a pyramid of images in which higher-level images in the pyramid are smaller and at lower resolution and lower-level images are larger and at higher resolution. Each layer of the pyramid is thus composed of small images, thereby allowing one to load only the pyramid level that is necessary to display or open for analysis. The next-generation file format (NGFF) has been developed by the OME team as a solution to the limitations of traditional image formats in microscopy (Moore et al. 2021a, 2021b). This file format is a multi-dimensional, multi-resolution, high-content pyramidal-image format that contains pixel data and metadata (such as annotations by a machine-learning tool). It is encouraged for data sharing and reuse, in particular for public data repositories and collaborative data resources. Importantly, NGFF is complementary to and does not supplant other open or proprietary formats because each image format has its own specification. For example, some image formats have optimised writing performance that is well suited to fast data capture whereas a machine-learning technology may depend on high-dimensional, high-content scalability to allow rich annotation of datasets (Moore et al. 2021a, 2021b).

### Operationalising the FAIR and CARE principles

That the big-data revolution in EM happens concomitantly with the growing importance of the FAIR and CARE principles (Wilkinson et al. 2016; Carroll et al. 2020) across the research sector offers the unique opportunity to tackle the consequences and challenges of the big-data deluge in a way that is sustainable and that maximises the output and impact of EM science.

The CARE Principles for Indigenous Data Governance are complementary to the FAIR principles. Whereas the FAIR principles propose a data-centric approach to data management, the CARE principles are people and purpose oriented (Carroll et al. 2021). Indigenous data are data, information and knowledge – in any format – that impact or concern Indigenous Peoples, nations and communities at the collective or individual levels. They encompass data about their resources and environments (Kukutai and Taylor 2016; Nickerson 2017). In the context of EM big data, it is important to be aware that it is possible that indigenous data may be buried in larger datasets, or that samples or additional data used in EM experiments or EM research projects may be associated with indigenous data. They may be hard to find, mislabelled and controlled by others in a manner inconsistent with the FAIR and CARE principles (Carroll et al. 2021). As a result, data could be subject to both CARE and FAIR. Given the tension between protecting Indigenous rights and interests in data while promoting FAIR data in research, the implementation of CARE should be considered as a required extra dimension of FAIR to ensure that the general use of data aligns with Indigenous rights. Importantly, researchers and facilities will find it easier to apply the CARE principles to data that are managed and properly documented through compliance with FAIR. Therefore, compliance with or promotion of CARE data is implied in the rest of the review when referring to FAIR data.

Putting FAIR and CARE into practice is associated with mainly three attributes of EM big data: vocabulary, visibility and value (Table 1). Advances in addressing or enhancing those three attributes contribute to enriching data to a higher FAIR or CARE state. This can have flow-on effects on the development and adoption of best practices in the management of EM big data overall, in particular in how big-data variety, veracity and volatility are dealt with.

The first step in reusing data is to find them. As shown earlier, data vocabulary through community-endorsed standards for data models together with data formats and metadata for the annotation and description of data is critical and integral to all aspects of FAIR (Wilkinson et al. 2016). In addition to accelerating the implementation and adoption of new standards, digital data repositories such as PDB and EMDB enhance data visibility. They provide stable, transparent, long-term storage and have become essential to the management and sharing of data (Habermann 2020). They increase the findability, accessibility, discoverability, sharing and reusability of data. They facilitate collaboration by creating an environment in which requesting and transferring data is easier. Researchers are encouraged to make their data available using repositories because data shared in repositories are more often cited than data shared by other means (i.e. data available on request, data contained within a publication and supplementary materials) (Colavizza et al. 2020). Importantly, FAIR metadata and data should be visible to, and easy to find for, both humans and computers. Thus, machine-readable metadata are essential for automatic and seamless discovery of data. The adoption of machine-actionable, community-endorsed, persistent identifiers (PIDs) plays an important role in findability, verification, replicability and reusability of data (Starr et al. 2015). PIDs are long-lasting, globally unique, digital references to objects, people or organisations. Digital Object Identifier (DOI) for resources (such as publications), persistent uniform resource locator (PURL) for web resources, ORCID ID for researchers and the Research Organization Registry (ROR) ID for research organisations (such as universities, centres and institutes) are well-established PIDs across all research communities. Additional PIDs are being developed so their levels of awareness and adoption can vary across communities. Initiatives that may be especially relevant to the biological EM community include the Research Resource Identifier (RRIDs) for antibodies, model organisms and tools such as software and databases (rrids.org) (Bandrowski et al. 2015), the International Generic Sample Number (IGSN) for physical samples (igsn.org), the Research Activity ID for research projects (raid.org.au) and PIDs for instrument. The latter follows recommendations by the Research Data Alliance Persistent Identification of Instruments Working Group (PIDINST) (Stocker et al. 2020). The work by the PIDINST group and the mapping of the schema developed by it onto the DataCite metadata schema (schema.datacite.org) emphasise the importance of instruments and associated metadata in the assessment of data quality and reuse. In Australia, Microscopy Australia is working towards a community-endorsed definition of instrument and guidelines for instrument PIDs. This is with the aim to promote and implement PIDs for all microscopes across Microscopy Australia’s research infrastructure facilities network. Instrument PIDs have many potential benefits such as the facilitation of asset management for a facility and the unambiguous reference to digital representations of instruments, including the generation of metrics that quantify the use of instruments and the rationale for future funding. Moreover, the linking of data to specific instruments enables citation and impact tracking. The benefits of such a rich ecosystem of PIDs can be further amplified by connecting them via their metadata in a PID graph (Cousijn et al. 2021). A PID graph establishes relationships between different entities within the research landscape (for example, objects, organisations, people, funders, instruments). This enables all stakeholders in research to access new information. For example, microscopy facilities can acquire a snapshot of the impact of the research that they have supported (such as number of publications, publication citations, cross-disciplinary impact and whether research outputs are reused). Overall, adopting PIDs and integrating them into platforms and information systems used in research organisations (e.g. research management, finances, human resources) facilitates information exchange between those systems across and between organisations. Critically, it eliminates the need to rekey information manually multiple times into multiple systems, for example about a grant, a publication or a person (e.g. publications, employment history), which leads to cost savings for research organisations (Brown et al. 2021, 2022).

Metadata and PIDs are fundamental in the operationalisation of the FAIR and CARE principles, thereby increasing the value of data. They increase trust in data and research reproducibility because they contain a range of information on the provenance of data and the tools, resources and methods associated with their creation before the experiment, during the experiment at the time of data capture and after the experiment during processing and analysis of data. Without thorough implementation of FAIR, the value of data peaks at publication and then falls off over time. Information entropy is the natural tendency for decline in data, information and understanding that occurs after data are used and results are published (Habermann 2020). Given the investment in big-data-producing electron microscopes, this is not a desired outcome. High-quality metadata that support understanding and reuse of data are a critical antidote to information entropy. A research data management plan that describes extensively how data will be annotated and described using metadata and PIDs and how this information will be obtained maximises the chance of high-value FAIR data (Michener 2015). Workflows that collect metadata reliably, consistently, systematically and automatically play an essential role in adding value to EM data. This requires scalable and adaptable software that integrates information from various sources into metadata, including electronic notebooks and instrument schedulers or booking systems at facilities. Besides widely used open-source applications for image data management such as OMERO (Allan et al. 2012; Burel et al. 2015; Li et al. 2016), the recently developed tools NexusLIMS (Taillon et al. 2021) and Pitschi (Nguyen 2022) are examples of data-workflow engines that assist in the capture and management of research data from electron microscopes as well as metadata from various sources (Table 2). Interestingly, both tools have been developed by microscopy research facilities with a focus on automated metadata harvest combined with an intuitive web-based GUI for searching, browsing and examining research data. In particular, Pitschi is an end-to-end data-management solution based on the Clowder framework (Marini et al. 2018) that supports the entire research data lifecycle by storing, indexing and annotating data generated at the facility from the capture of raw data at the instruments (Nguyen 2022). Pitschi is fully integrated with the data storage infrastructure at The University of Queensland (Brisbane, Australia) where it has been developed. Importantly, Pitschi adheres to and fosters the FAIR principles. Metadata of supported file types are extracted automatically during data ingestion. There is also the option to enrich metadata. For example, information such as users’ ORCID IDs and instrument PIDs can be collected into metadata from a range of sources such as the instrument-booking system of the facility.

Finally, it is important to note that operationalising FAIR and CARE implies paying attention that platforms such as VREs used for data processing, analysis and visualisation enable FAIR data and enrich data that they create to a richer FAIR state (or, at least, not make them less FAIR than input data). Despite their advantages, some platforms, in particular cloud ones, do not automatically or natively support FAIR. Cloud platforms tend to encourage self-sufficient environments including data repositories at the expense of interoperable services within and outside the cloud (Sheffield et al. 2022). The Research Data Alliance FAIR for VREs Working Group (rd-alliance.org/groups/fair-virtual-research-environments) is developing guidelines to ensure that VREs enable FAIR data in coordination with existing communities working with VREs and VRE developers.

## Conclusions and future perspectives

The big-data revolution undergone by electron microscopy (EM) presents the opportunity to maximise the value of the investment in EM and EM data themselves by implementing approaches to transfer, compute and manage big data in ways that are faster, more accessible, more reliable and more sustainable. While the ten V’s of EM big data (volume, variety, velocity, veracity, value, visibility, visualisation, vocabulary, variability and volatility) have created challenges for researchers and microscopy research facilities, each challenge is a chance for optimised workflows, greater research impact, richer metadata or widely adopted best practices. This review highlights an overall need for more or better engagement and coordination across the EM community in two areas: first, in the sharing of experiences on how to adapt and optimise EM-underpinning infrastructure to big-data transfer, processing and analysis; and secondly, in the establishment and fostering of guidelines, standards and conventions for the development of VREs, the unification of data formats (or rationalisation in their numbers) and the collection of metadata for data description and annotation. Both aspects are especially important as the integration of omics and EM into single, multi-modal characterisation approaches will lead to even larger and more diverse datasets generated in high throughput (McCafferty et al. 2020; Kuhn Cuellar et al. 2022; Watson et al. 2022). Finally, the strong drive to make scientific data FAIR and CARE is a golden opportunity for the microscopy community because it emphasises that standards for data and metadata cannot be defined by individual laboratories, research groups or microscope manufacturers for reasons that suit their own needs and interests. Instead, they should arise from extensive, community-wide consultations to ensure rapid adoption that will serve the science community for the foreseeable future to come.

## Data Availability

Data sharing is not applicable to this review as no dataset was generated or analysed during the current study.
